# Supplementary material for: Chromothripsis during telomere crisis is independent of NHEJ, and consistent with a replicative origin
Source: Genome Res. 2019 May;29(5):737–49. doi: 10.1101/gr.240705.118 (PMC6499312; doi:10.1101/gr.240705.118)
Supplement: Supplemental Material [file supp_gr.240705.118_Supplemental_file_1.zip › contigs/annotated_contigs/DB53/contig.3.DB53_length_529_mean_cov_10.561436673.docx]

**DB53_length_529_mean_cov_10.561436673**

```
TTTTTTACTTTTTTTTGTGTTTTTTATTTCGCATTATTTTTTTCAATTC|TTTTAATTTTATCCTTTGTGCTTTTTTT|TAATTAATCC
                                                  >chr17:66668862-66668890 + E=3e-02
CTGAGTTCTTTGTAAATATGCTTTTTGTTTAAC|TTTTTTTTTTTGAGATGGAGTCTCACTCTGTCACCCAGGCTGGAGTGCAGTGGCG
                                  >chr2:173401516-173401682 + E=3e-64
TGATCTCGGCTCACTGCAAGCTCCGCCTCCTGGGTTCCCACCATTCTCCTGCCTCAGCCTCCCGAGTAGCTGGGACAACAGGAGTCCAC

CACCACGCCCGGCTAATTTTTT|T|TTATTCTCTTCTCCAACCTCTCTCACTATCCCTCAGCTTATTTCTCCTTTCAATTTCTGCACCA
                        >chr8:129927277-129927520 + E=1e-84
CCCTTCAATCTCTCCGTTCTTTTAATTTCAATTCCTTTCCTTTTCTGGTAGAGAAGGAGACGCGTTTTATCTGTGGACCCAAAACTCCG

GCGCCGGTCACGGACTTAGGAAGGCAGCCTTCCCTTGGTGTTTAATCATTGCAGGGACGCTTCTCTGATTTTTCACCCATGTTTCAGAG
```
